# 5-Dimethyl­amino-*N*,*N*-dimethyl-2-nitro­benzamide

**DOI:** 10.1107/S1600536809018017

**Published:** 2009-05-20

**Authors:** Hoong-Kun Fun, Samuel Robinson Jebas, B. Chandrakantha, Vijaya Padmar, Arun M Isloor

**Affiliations:** a X-ray Crystallography Unit, School of Physics, Universiti Sains Malaysia, 11800 USM, Penang, Malaysia; b Manipal Institute of Technology, Manipal University, Manipal 576 104, India; c Department of Chemistry, National Institute of Technology-Karnataka, Surathkal, Mangalore 575 025, India

## Abstract

In the title compound, C_11_H_15_N_3_O_3_, one of the methyl groups attached to the benzamide unit is slightly twisted with a C—N—C—C torsion angle of 4.04 (13)°. The crystal packing is stabilized by weak intermolecular C—H⋯O hydrogen bonds together with a weak C—H⋯π inter­action.

## Related literature

For nitro­aniline mustards, see: Brian *et al.* (1992 ▶); Rauth, (1984 ▶). For *N*-[(*N*,*N*-dimethyl­amino)eth­yl]carboxamide derivatives, see: Alston *et al.* (1983 ▶); Denny & Wilson (1986 ▶); Palmer *et al.* (1990 ▶). For bond-length data, see: Allen *et al.* (1987 ▶). For the stability of the temperature controller used in the data collection, see: Cosier & Glazer (1986 ▶).
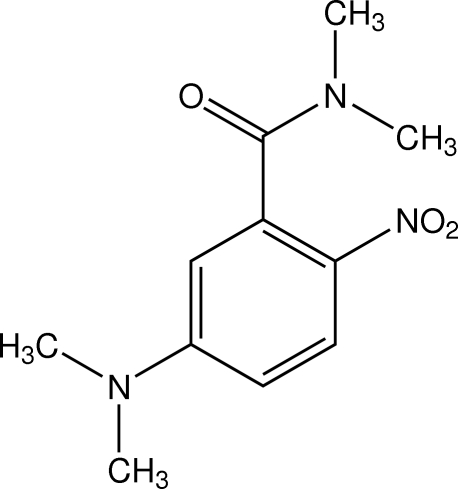


## Experimental

### 

#### Crystal data

C_11_H_15_N_3_O_3_
*M* *_r_* = 237.26
Monoclinic, 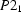

*a* = 7.8581 (2) Å
*b* = 7.2921 (2) Å
*c* = 10.5183 (3) Å
β = 104.663 (1)°
*V* = 583.09 (3) Å^3^
*Z* = 2
Mo *K*α radiation
μ = 0.10 mm^−1^
*T* = 100 K
0.52 × 0.45 × 0.33 mm

#### Data collection

Bruker SMART APEXII CCD area-detector diffractometer
Absorption correction: multi-scan (*SADABS*; Bruker, 2005 ▶) *T*_min_ = 0.949, *T*_max_ = 0.965
16075 measured reflections
3233 independent reflections
3002 reflections with *I* > 2σ(*I*)
*R*_int_ = 0.027

#### Refinement

*R*[*F* ^2^ > 2σ(*F* ^2^)] = 0.038
*wR*(*F* ^2^) = 0.103
*S* = 1.08
3233 reflections
158 parameters
1 restraint
H-atom parameters constrained
Δρ_max_ = 0.64 e Å^−3^
Δρ_min_ = −0.19 e Å^−3^

### 

Data collection: *APEX2* (Bruker, 2005 ▶); cell refinement: *SAINT* (Bruker, 2005 ▶); data reduction: *SAINT*; program(s) used to solve structure: *SHELXTL* (Sheldrick, 2008 ▶); program(s) used to refine structure: *SHELXTL*; molecular graphics: *SHELXTL*; software used to prepare material for publication: *SHELXTL* and *PLATON* (Spek, 2009 ▶).

## Supplementary Material

Crystal structure: contains datablocks global, I. DOI: 10.1107/S1600536809018017/dn2453sup1.cif

Structure factors: contains datablocks I. DOI: 10.1107/S1600536809018017/dn2453Isup2.hkl

Additional supplementary materials:  crystallographic information; 3D view; checkCIF report

## Figures and Tables

**Table 1:** Hydrogen-bond geometry (Å, °)

*D*—H⋯*A*	*D*—H	H⋯*A*	*D*⋯*A*	*D*—H⋯*A*
C8—H8*A*⋯O3^i^	0.96	2.52	3.1751 (16)	126
C10—H10*A*⋯O2^ii^	0.96	2.59	3.5413 (14)	173
C10—H10*B*⋯O1^iii^	0.96	2.49	3.3920 (13)	156
C4—H4*A*⋯*Cg*1^iv^	0.93	2.81	3.3991 (11)	122

Symmetry codes: (i) 
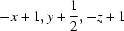
; (ii) 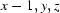; (iii) 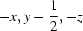; (iv) 
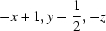
. *Cg*1 is the centroid of the C1–C6 ring.

**Fig. 1.**
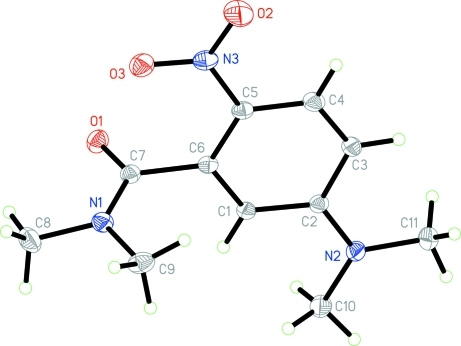
The molecular structure of the title compound, showing 50% probability displacement ellipsoids and the atom numbering scheme.

**Fig. 2.**
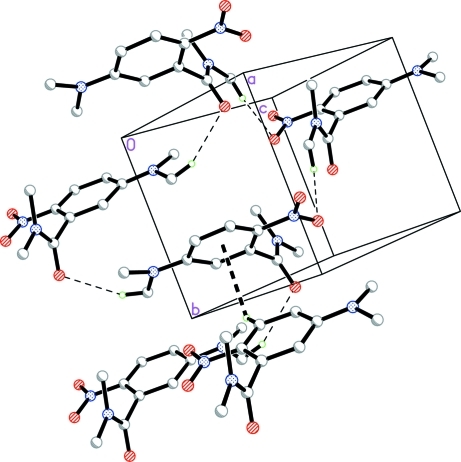
The crystal packing of the title compound, viewed along the a axis. Dashed lines indicate the hydrogen bonding and C-H···π interactions.

## supplementary crystallographic information

### Comment

Nitroaniline mustards are potential hypoxia-selective cytotoxic agents (Rauth, 1984) possessing reductive metabolism which activates the mustard nitrogen by converting the electron-withdrawing nitro group to an electron-donating hydroxylamine or amine (Brian *et al.*, 1992). *N*-[(*N*,*N*-dimethylamino)ethyl]carboxamide derivatives proved to have excellent aqueous solubility and improved cytotoxic potency (Denny *et al.*, 1986), but their reduction potentials, while higher than the non carboxamide compounds, were still low and have limited selectivity for hypoxic cells. (Palmer *et al.*, 1990; Alston *et al.*, 1983). These properties prompted us to synthesize the title compound.

In the asymmetric unit of (I), (Fig 1), one of the methyl group attached to the benzamide unit is slightly twisted as indicated by the torsion angle of C9–N1–C7–C6=4.04 (13)°. The benzene ring is essentially planar with the maximum deviation from planarity of 0.0117 (10)Å for the atom C5. The bond lengths and bond angles are normal (Allen *et al.*, 1987).

The crystal packing is stabilized by intermolecular weak C—H···O hydrogen bonds together with weak C—H···π interaction (Table 1, Fig. 2).

### Experimental

5-Fluoro-2-nitrobenzoic acid (5 g, 0.0270 mol) was heated with 40% aqueous dimethyl amine solution (50 ml) at 100°C for 18 hrs. Reaction mixture was concentrated through rotovap using high vacuum pump to afford 5-(dimethylamino)-2-nitrobenzoic acid as yellow crystalline solid (4.5 g). To a solution of 5-(dimethylamino)-2-nitrobenzoic acid (4 g, 0.019 mol) in DMF(40 ml) was added 1-(3-Dimethylaminopropyl)-3-ethyl carbodiimide hydrochloride (5.4 g, 0.0285 mol), 1-hydroxy benzotriazole (0.25 g, 0.0019 mol) and *N*,*N*-diisopropyl ethylamine (4.9 g, 0.038 mol) and the reaction mixture was stirred at room temperature for 18 hrs. Reaction mixture was portioned between 10% sodium bicarbonate solution and ethyl acetate (25 ml). The organic layer was washed with brine and dried over anhydrous sodium sulfate and concentrated through rotovap. The concentrated product were purified by column chromatography (60–120 mesh silica gel) using hexane and ethyl acetate as eluent to afford 5-(dimethylamino)-N, *N*-dimethyl-2-nitrobenzamide (3 g, 66.6%) as yellow crystalline solid. *M*.p was found to be 458–461 K.

### Refinement

H atoms were positioned geometrically [C–H = 0.93–0.96 Å] and refined using a riding model, with *U*_iso_(H) = 1.2*U*_eq_(C) and 1.5*U*_eq_(methyl C). A rotating–group model was used for the methyl groups. Since there is no anamolous scattering effects, 2707 Friedel pairs were merged before final refinement.

The largest residual electron densities are located in the middle of some C-C bonds within the phenyl ring.

### Crystal data

**Table:**

C_11_H_15_N_3_O_3_	*F*(000) = 252
*M**_r_* = 237.26	*D*_x_ = 1.351 Mg m^−^^3^
Monoclinic, *P*2_1_	Mo *K*α radiation, λ = 0.71073 Å
Hall symbol: P 2yb	Cell parameters from 9204 reflections
*a* = 7.8581 (2) Å	θ = 2.7–40.5°
*b* = 7.2921 (2) Å	µ = 0.10 mm^−^^1^
*c* = 10.5183 (3) Å	*T* = 100 K
β = 104.663 (1)°	Block, yellow
*V* = 583.09 (3) Å^3^	0.52 × 0.45 × 0.33 mm
*Z* = 2	

### Data collection

**Table:**

Bruker SMART APEXII CCD area-detector diffractometer	3233 independent reflections
Radiation source: fine-focus sealed tube	3002 reflections with *I* > 2σ(*I*)
graphite	*R*_int_ = 0.027
φ and ω scans	θ_max_ = 37.5°, θ_min_ = 2.7°
Absorption correction: multi-scan (*SADABS*; Bruker, 2005)	*h* = −13→13
*T*_min_ = 0.949, *T*_max_ = 0.965	*k* = −12→12
16075 measured reflections	*l* = −18→18

### Refinement

**Table:**

Refinement on *F*^2^	Primary atom site location: structure-invariant direct methods
Least-squares matrix: full	Secondary atom site location: difference Fourier map
*R*[*F*^2^ > 2σ(*F*^2^)] = 0.038	Hydrogen site location: inferred from neighbouring sites
*wR*(*F*^2^) = 0.103	H-atom parameters constrained
*S* = 1.08	*w* = 1/[σ^2^(*F*_o_^2^) + (0.0716*P*)^2^ + 0.0071*P*] where *P* = (*F*_o_^2^ + 2*F*_c_^2^)/3
3233 reflections	(Δ/σ)_max_ < 0.001
158 parameters	Δρ_max_ = 0.64 e Å^−^^3^
1 restraint	Δρ_min_ = −0.19 e Å^−^^3^

### Special details

**Table:**

Experimental. The crystal was placed in the cold stream of an Oxford Cyrosystems Cobra open-flow nitrogen cryostat (Cosier & Glazer, 1986) operating at 100.0 (1) K.
Geometry. All e.s.d.'s (except the e.s.d. in the dihedral angle between two l.s. planes) are estimated using the full covariance matrix. The cell e.s.d.'s are taken into account individually in the estimation of e.s.d.'s in distances, angles and torsion angles; correlations between e.s.d.'s in cell parameters are only used when they are defined by crystal symmetry. An approximate (isotropic) treatment of cell e.s.d.'s is used for estimating e.s.d.'s involving l.s. planes.
Refinement. Refinement of *F*^2^ against ALL reflections. The weighted *R*-factor *wR* and goodness of fit *S* are based on *F*^2^, conventional *R*-factors *R* are based on *F*, with *F* set to zero for negative *F*^2^. The threshold expression of *F*^2^ > σ(*F*^2^) is used only for calculating *R*-factors(gt) *etc*. and is not relevant to the choice of reflections for refinement. *R*-factors based on *F*^2^ are statistically about twice as large as those based on *F*, and *R*- factors based on ALL data will be even larger.

### Fractional atomic coordinates and isotropic or equivalent isotropic displacement parameters (Å^2^)

**Table:**

	*x*	*y*	*z*	*U*_iso_*/*U*_eq_	
O1	0.42663 (10)	1.04274 (10)	0.31204 (7)	0.01969 (13)	
O2	0.81992 (10)	0.62755 (17)	0.20077 (10)	0.0334 (2)	
O3	0.71314 (10)	0.76718 (13)	0.34531 (8)	0.02322 (15)	
N1	0.32548 (10)	0.78907 (13)	0.39646 (8)	0.01811 (14)	
N2	0.03825 (10)	0.70383 (12)	−0.13123 (8)	0.01594 (13)	
N3	0.69532 (10)	0.69824 (13)	0.23538 (9)	0.01899 (15)	
C1	0.21826 (10)	0.78067 (12)	0.08485 (8)	0.01443 (13)	
H1A	0.1210	0.8309	0.1072	0.017*	
C2	0.19805 (10)	0.70835 (12)	−0.04364 (9)	0.01396 (13)	
C3	0.35014 (11)	0.63797 (13)	−0.07614 (9)	0.01687 (15)	
H3A	0.3427	0.5950	−0.1606	0.020*	
C4	0.50889 (12)	0.63311 (14)	0.01717 (10)	0.01789 (16)	
H4A	0.6070	0.5837	−0.0045	0.021*	
C5	0.52463 (11)	0.70133 (13)	0.14373 (9)	0.01538 (14)	
C6	0.37880 (10)	0.77846 (12)	0.17780 (8)	0.01392 (13)	
C7	0.38381 (11)	0.87931 (13)	0.30423 (8)	0.01530 (14)	
C8	0.30516 (14)	0.88622 (18)	0.51258 (10)	0.02438 (19)	
H8A	0.3409	1.0116	0.5088	0.037*	
H8B	0.3770	0.8290	0.5899	0.037*	
H8C	0.1842	0.8822	0.5156	0.037*	
C9	0.26858 (13)	0.59813 (16)	0.38621 (11)	0.02241 (18)	
H9A	0.3149	0.5368	0.3215	0.034*	
H9B	0.1424	0.5930	0.3606	0.034*	
H9C	0.3109	0.5389	0.4698	0.034*	
C10	−0.11334 (12)	0.79019 (15)	−0.10016 (10)	0.02084 (17)	
H10A	−0.1220	0.7503	−0.0151	0.031*	
H10B	−0.2183	0.7562	−0.1650	0.031*	
H10C	−0.0997	0.9210	−0.0999	0.031*	
C11	0.02302 (13)	0.65472 (17)	−0.26808 (10)	0.02261 (18)	
H11A	0.0664	0.5324	−0.2724	0.034*	
H11B	0.0908	0.7388	−0.3056	0.034*	
H11C	−0.0982	0.6607	−0.3163	0.034*	

### Atomic displacement parameters (Å^2^)

**Table:**

	*U*^11^	*U*^22^	*U*^33^	*U*^12^	*U*^13^	*U*^23^
O1	0.0221 (3)	0.0166 (3)	0.0198 (3)	−0.0026 (2)	0.0044 (2)	−0.0016 (2)
O2	0.0152 (3)	0.0482 (6)	0.0356 (4)	0.0109 (3)	0.0039 (3)	−0.0048 (4)
O3	0.0180 (3)	0.0286 (4)	0.0212 (3)	−0.0001 (3)	0.0016 (2)	0.0009 (3)
N1	0.0175 (3)	0.0211 (4)	0.0163 (3)	−0.0025 (3)	0.0053 (2)	0.0010 (3)
N2	0.0142 (3)	0.0169 (3)	0.0162 (3)	0.0002 (2)	0.0029 (2)	−0.0020 (3)
N3	0.0132 (3)	0.0199 (3)	0.0232 (3)	0.0010 (3)	0.0033 (2)	0.0028 (3)
C1	0.0119 (3)	0.0156 (3)	0.0163 (3)	0.0001 (2)	0.0045 (2)	−0.0015 (3)
C2	0.0134 (3)	0.0125 (3)	0.0162 (3)	−0.0006 (2)	0.0042 (2)	−0.0005 (3)
C3	0.0154 (3)	0.0165 (3)	0.0198 (4)	0.0002 (3)	0.0065 (3)	−0.0033 (3)
C4	0.0142 (3)	0.0175 (4)	0.0230 (4)	0.0013 (3)	0.0065 (3)	−0.0022 (3)
C5	0.0117 (3)	0.0151 (3)	0.0191 (3)	0.0008 (3)	0.0036 (2)	0.0010 (3)
C6	0.0128 (3)	0.0134 (3)	0.0159 (3)	−0.0007 (2)	0.0043 (2)	0.0011 (3)
C7	0.0131 (3)	0.0175 (3)	0.0150 (3)	−0.0004 (3)	0.0029 (2)	0.0003 (3)
C8	0.0253 (4)	0.0322 (5)	0.0174 (4)	0.0020 (4)	0.0086 (3)	0.0002 (4)
C9	0.0205 (4)	0.0228 (4)	0.0232 (4)	−0.0045 (3)	0.0041 (3)	0.0059 (3)
C10	0.0145 (3)	0.0253 (4)	0.0214 (4)	0.0033 (3)	0.0020 (3)	−0.0045 (4)
C11	0.0208 (4)	0.0303 (5)	0.0164 (3)	−0.0019 (3)	0.0040 (3)	−0.0045 (3)

### Geometric parameters (Å, °)

**Table:**

O1—C7	1.2354 (12)	C4—C5	1.3969 (13)
O2—N3	1.2400 (12)	C4—H4A	0.9300
O3—N3	1.2358 (12)	C5—C6	1.4021 (11)
N1—C7	1.3450 (12)	C6—C7	1.5115 (13)
N1—C8	1.4553 (13)	C8—H8A	0.9600
N1—C9	1.4580 (14)	C8—H8B	0.9600
N2—C2	1.3569 (11)	C8—H8C	0.9600
N2—C10	1.4558 (12)	C9—H9A	0.9600
N2—C11	1.4585 (12)	C9—H9B	0.9600
N3—C5	1.4403 (12)	C9—H9C	0.9600
C1—C6	1.3868 (11)	C10—H10A	0.9600
C1—C2	1.4216 (12)	C10—H10B	0.9600
C1—H1A	0.9300	C10—H10C	0.9600
C2—C3	1.4197 (11)	C11—H11A	0.9600
C3—C4	1.3787 (12)	C11—H11B	0.9600
C3—H3A	0.9300	C11—H11C	0.9600
			
C7—N1—C8	119.80 (9)	O1—C7—N1	124.11 (9)
C7—N1—C9	124.61 (8)	O1—C7—C6	118.29 (8)
C8—N1—C9	115.48 (8)	N1—C7—C6	117.31 (8)
C2—N2—C10	120.41 (7)	N1—C8—H8A	109.5
C2—N2—C11	120.45 (7)	N1—C8—H8B	109.5
C10—N2—C11	117.41 (8)	H8A—C8—H8B	109.5
O3—N3—O2	122.21 (9)	N1—C8—H8C	109.5
O3—N3—C5	119.06 (8)	H8A—C8—H8C	109.5
O2—N3—C5	118.74 (9)	H8B—C8—H8C	109.5
C6—C1—C2	121.92 (7)	N1—C9—H9A	109.5
C6—C1—H1A	119.0	N1—C9—H9B	109.5
C2—C1—H1A	119.0	H9A—C9—H9B	109.5
N2—C2—C3	121.23 (8)	N1—C9—H9C	109.5
N2—C2—C1	121.07 (7)	H9A—C9—H9C	109.5
C3—C2—C1	117.69 (7)	H9B—C9—H9C	109.5
C4—C3—C2	120.20 (8)	N2—C10—H10A	109.5
C4—C3—H3A	119.9	N2—C10—H10B	109.5
C2—C3—H3A	119.9	H10A—C10—H10B	109.5
C3—C4—C5	121.00 (8)	N2—C10—H10C	109.5
C3—C4—H4A	119.5	H10A—C10—H10C	109.5
C5—C4—H4A	119.5	H10B—C10—H10C	109.5
C4—C5—C6	120.36 (8)	N2—C11—H11A	109.5
C4—C5—N3	118.37 (7)	N2—C11—H11B	109.5
C6—C5—N3	121.20 (8)	H11A—C11—H11B	109.5
C1—C6—C5	118.75 (8)	N2—C11—H11C	109.5
C1—C6—C7	115.54 (7)	H11A—C11—H11C	109.5
C5—C6—C7	125.39 (7)	H11B—C11—H11C	109.5
			
C10—N2—C2—C3	−174.89 (9)	C2—C1—C6—C5	−0.44 (13)
C11—N2—C2—C3	−10.25 (14)	C2—C1—C6—C7	173.36 (8)
C10—N2—C2—C1	6.32 (14)	C4—C5—C6—C1	1.86 (13)
C11—N2—C2—C1	170.96 (9)	N3—C5—C6—C1	178.85 (8)
C6—C1—C2—N2	176.85 (9)	C4—C5—C6—C7	−171.28 (9)
C6—C1—C2—C3	−1.98 (13)	N3—C5—C6—C7	5.71 (14)
N2—C2—C3—C4	−175.78 (9)	C8—N1—C7—O1	1.74 (14)
C1—C2—C3—C4	3.05 (13)	C9—N1—C7—O1	177.67 (9)
C2—C3—C4—C5	−1.73 (15)	C8—N1—C7—C6	−171.90 (8)
C3—C4—C5—C6	−0.79 (15)	C9—N1—C7—C6	4.04 (13)
C3—C4—C5—N3	−177.86 (9)	C1—C6—C7—O1	−91.10 (10)
O3—N3—C5—C4	176.11 (9)	C5—C6—C7—O1	82.24 (12)
O2—N3—C5—C4	−3.35 (14)	C1—C6—C7—N1	82.91 (10)
O3—N3—C5—C6	−0.94 (14)	C5—C6—C7—N1	−103.75 (10)
O2—N3—C5—C6	179.61 (10)		

### Hydrogen-bond geometry (Å, °)

**Table:**

*D*—H···*A*	*D*—H	H···*A*	*D*···*A*	*D*—H···*A*
C8—H8A···O3^i^	0.96	2.52	3.1751 (16)	126
C10—H10A···O2^ii^	0.96	2.59	3.5413 (14)	173
C10—H10B···O1^iii^	0.96	2.49	3.3920 (13)	156
C4—H4A···Cg1^iv^	0.93	2.81	3.3991 (11)	122

Symmetry codes: (i) −*x*+1, *y*+1/2, −*z*+1; (ii) *x*−1, *y*, *z*; (iii) −*x*, *y*−1/2, −*z*; (iv) −*x*+1, *y*−1/2, −*z*.

## References

[bb1] Allen, F. H., Kennard, O., Watson, D. G., Brammer, L., Orpen, A. G. & Taylor, R. (1987). *J. Chem. Soc. Perkin Trans. 2*, pp. S1–19.

[bb2] Alston, T. A., Porter, D. J. & Bright, H. J. (1983). *Acc. Chem. Res.* **16**, 418–442.

[bb3] Brian, D. P., William, R. W., Stephen, C. & William, A. D. (1992). *J. Med. Chem.* **35**, 3214–3222.

[bb4] Bruker (2005). *APEX2*, *SAINT* and *SADABS* Bruker AXS Inc., Madison, Wisconsin, USA.

[bb5] Cosier, J. & Glazer, A. M. (1986). *J. Appl. Cryst.* **19**, 105–107.

[bb6] Denny, W. A. & Wilson, W. R. (1986). *J. Med. Chem.* **29**, 879–887. 10.1021/jm00156a001 3712377

[bb7] Palmer, B. D., Wilson, W. R., Pullen, S. M. & Denny, W. A. (1990). *J. Med. Chem.* **33**, 112–121. 10.1021/jm00163a019 2296009

[bb8] Rauth, A. M. (1984). *Oncol. Biol. Phys.* **10**, 1293–1300. 10.1016/0360-3016(84)90335-3 6469753

[bb9] Sheldrick, G. M. (2008). *Acta Cryst.* A**64**, 112–122. 10.1107/S0108767307043930 18156677

[bb10] Spek, A. L. (2009). *Acta Cryst.* D**65**, 148–155. 10.1107/S090744490804362X PMC2631630 19171970

